# Supplementary figures and images for: Discrete natural neighbour interpolation with uncertainty using cross-validation error-distance fields
Source: PeerJ Comput Sci. 2020 Jul 13;6:e282. doi: 10.7717/peerj-cs.282 (PMC7924714; doi:10.7717/peerj-cs.282)

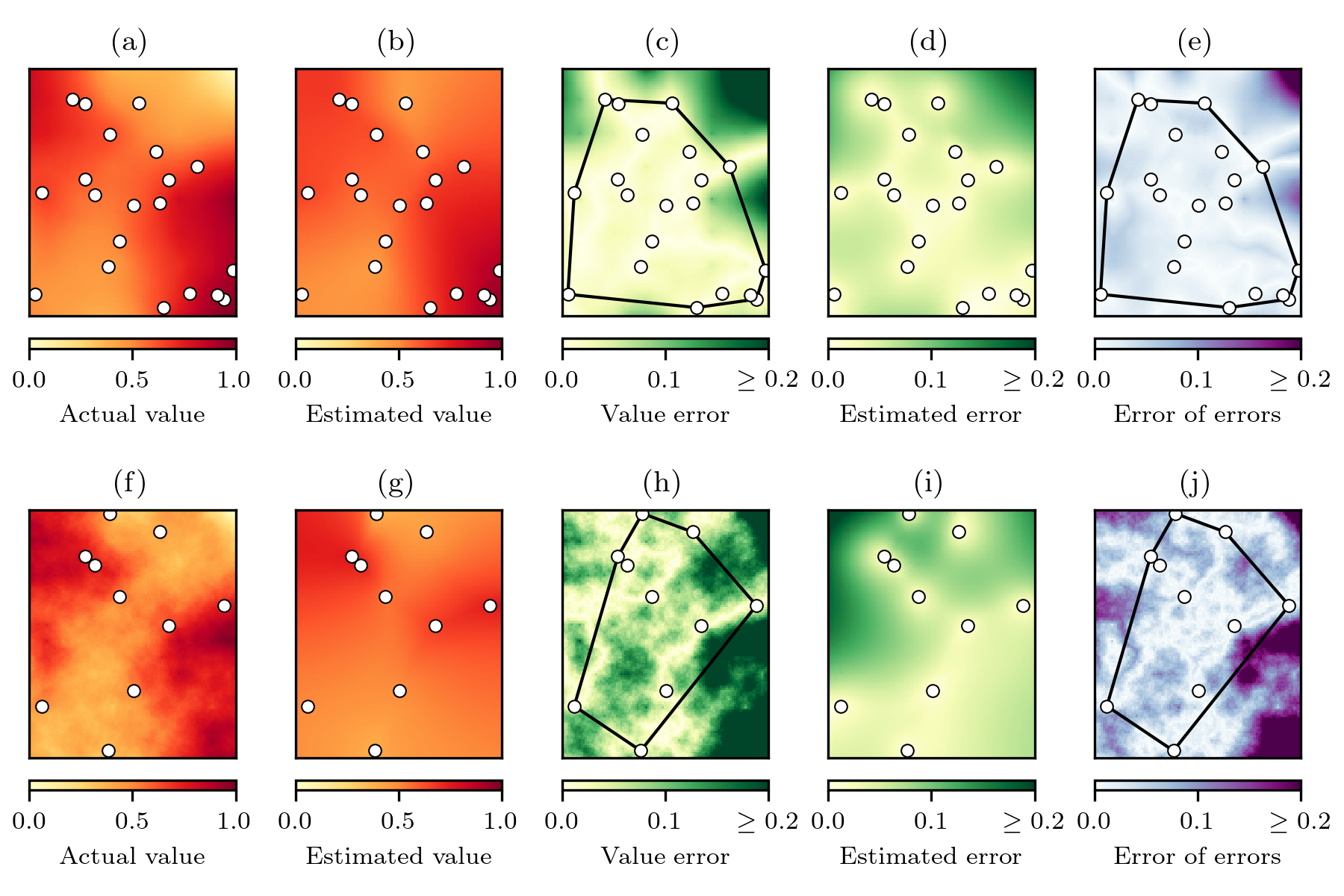

Supplement: Supplemental Information 1 [file peerj-cs-06-282-s001.zip › experimental-process/figure-5.png]

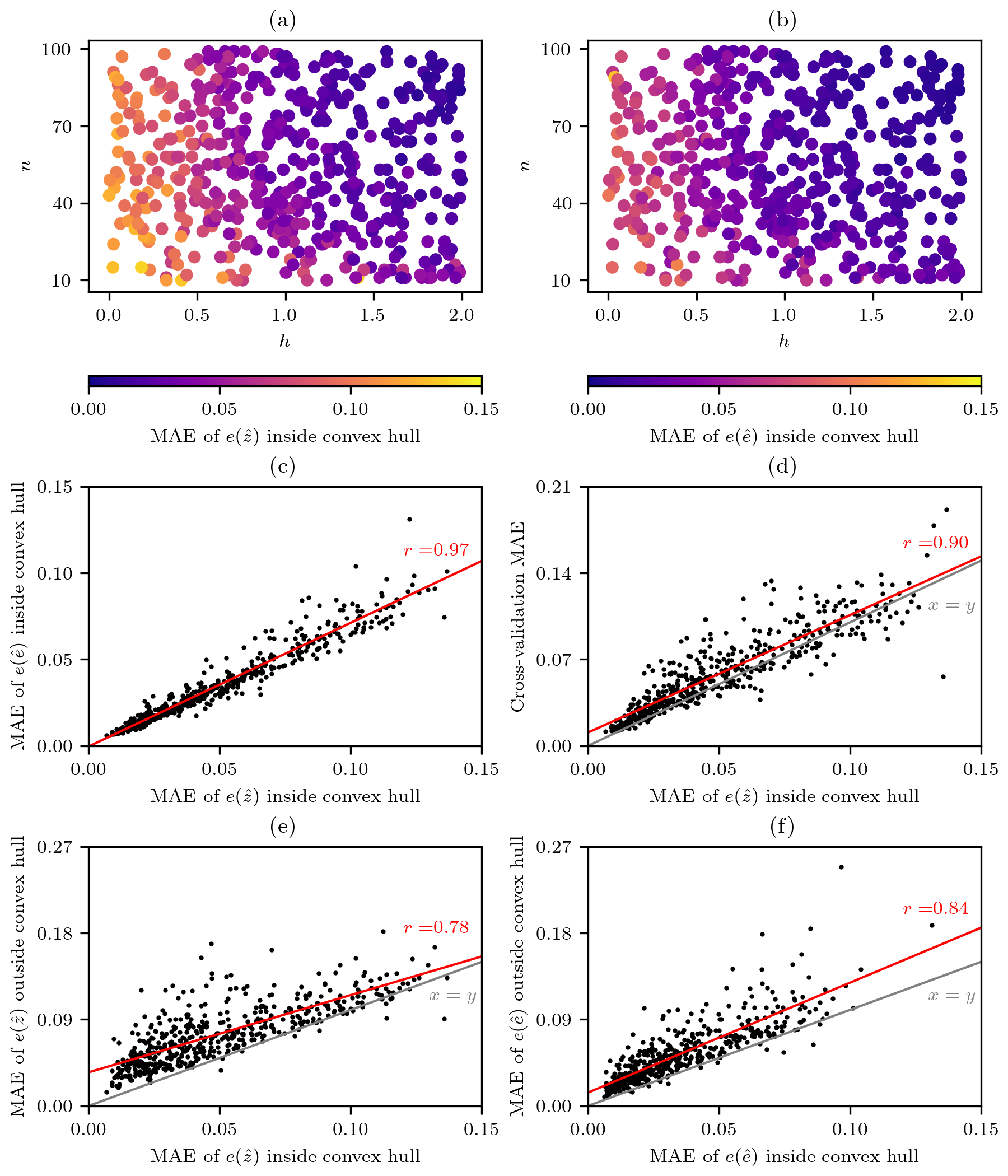

Supplement: Supplemental Information 1 [file peerj-cs-06-282-s001.zip › experiments/figure-6.png]

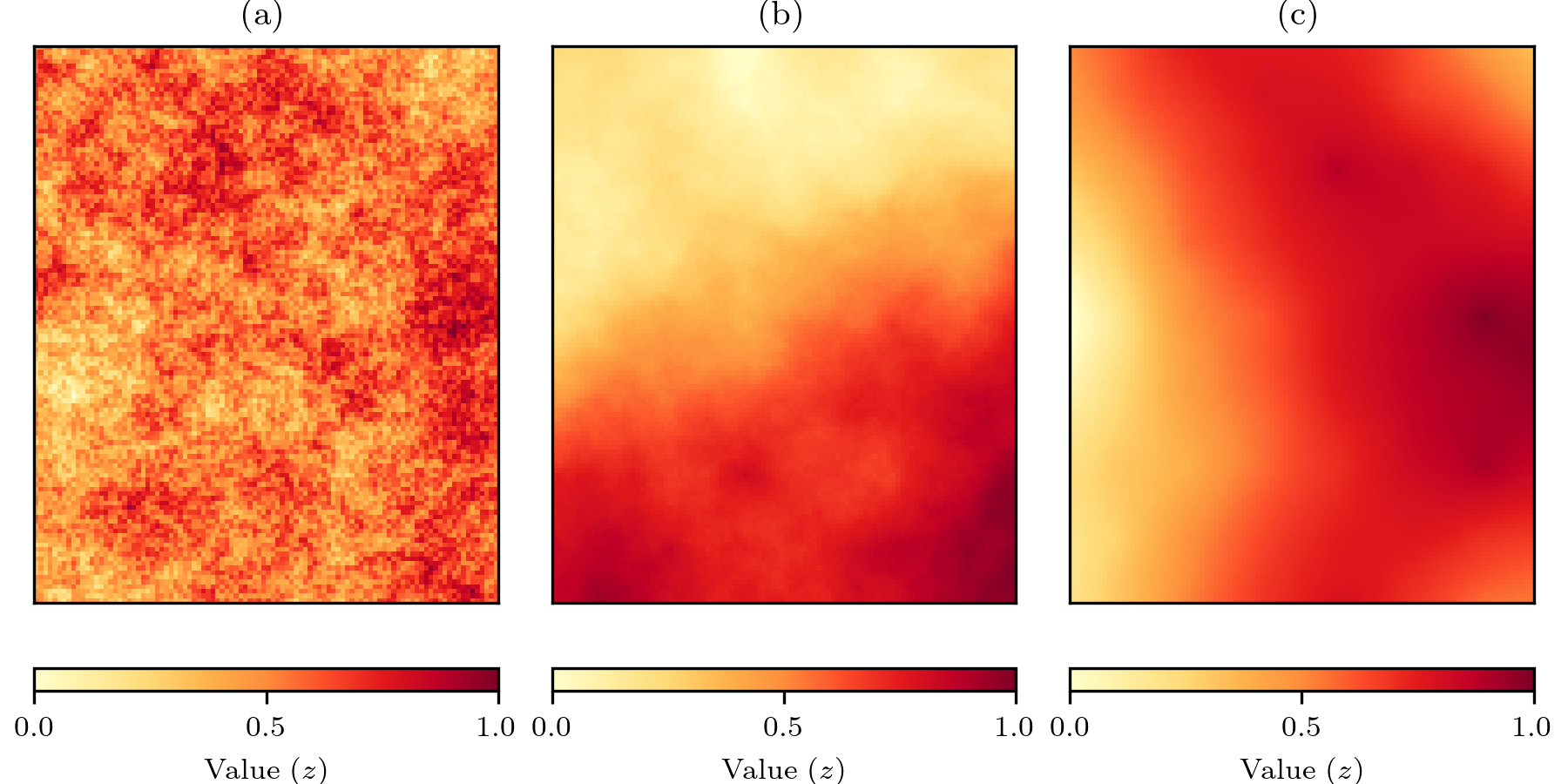

Supplement: Supplemental Information 1 [file peerj-cs-06-282-s001.zip › nlms/figure-4.png]

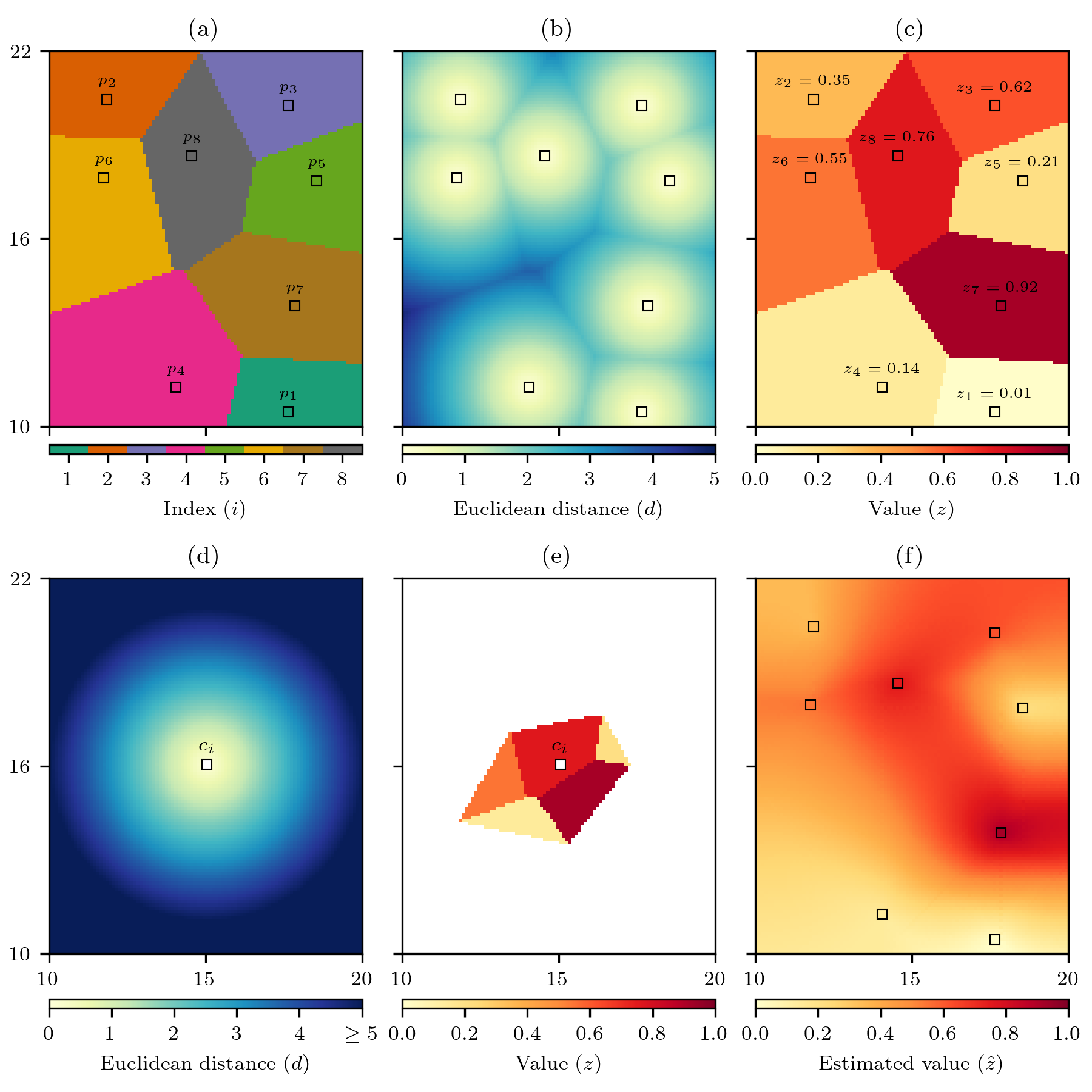

Supplement: Supplemental Information 1 [file peerj-cs-06-282-s001.zip › nni-discrete-method/figure-1.png]

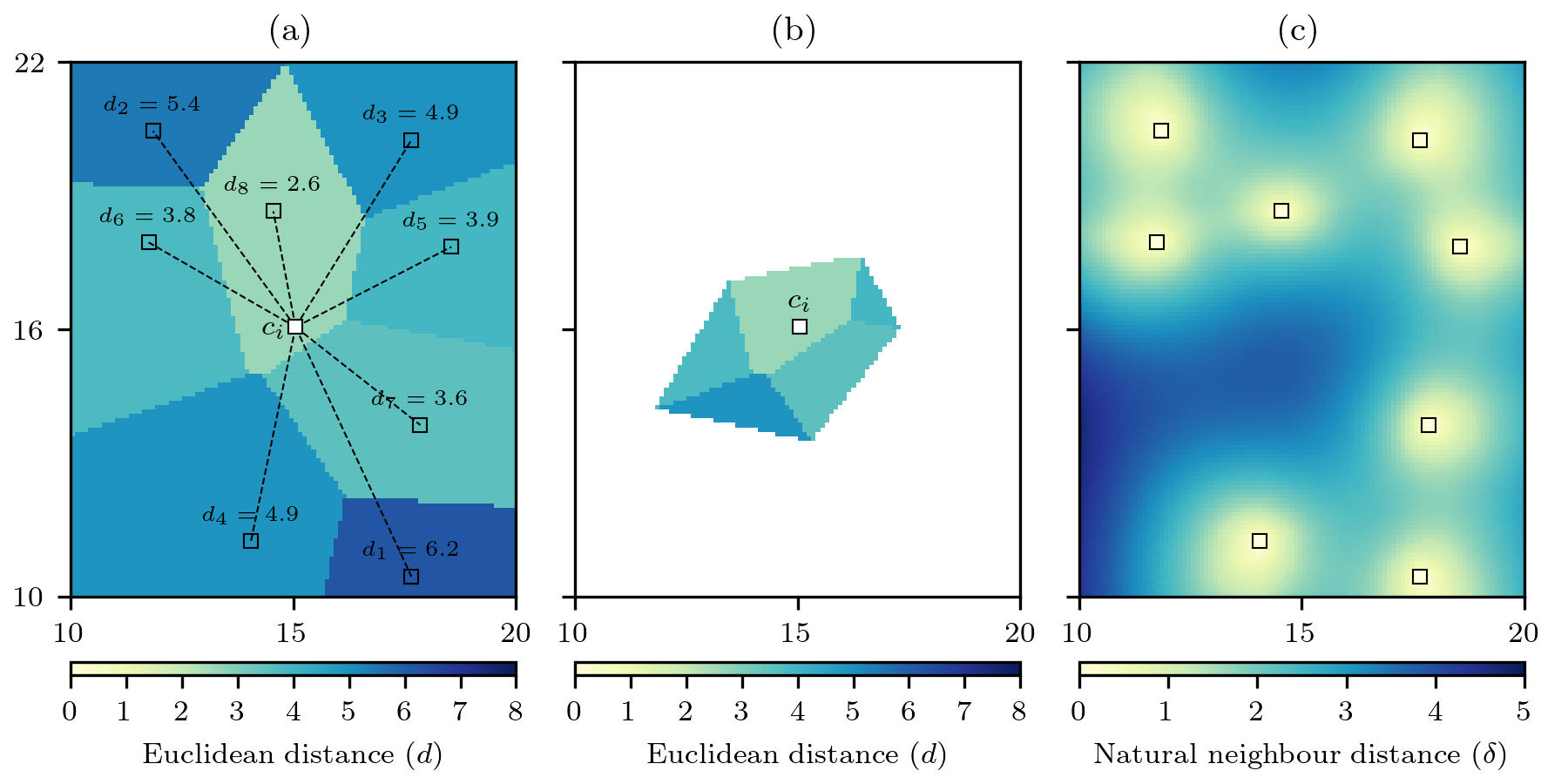

Supplement: Supplemental Information 1 [file peerj-cs-06-282-s001.zip › nni-discrete-method/figure-2.png]

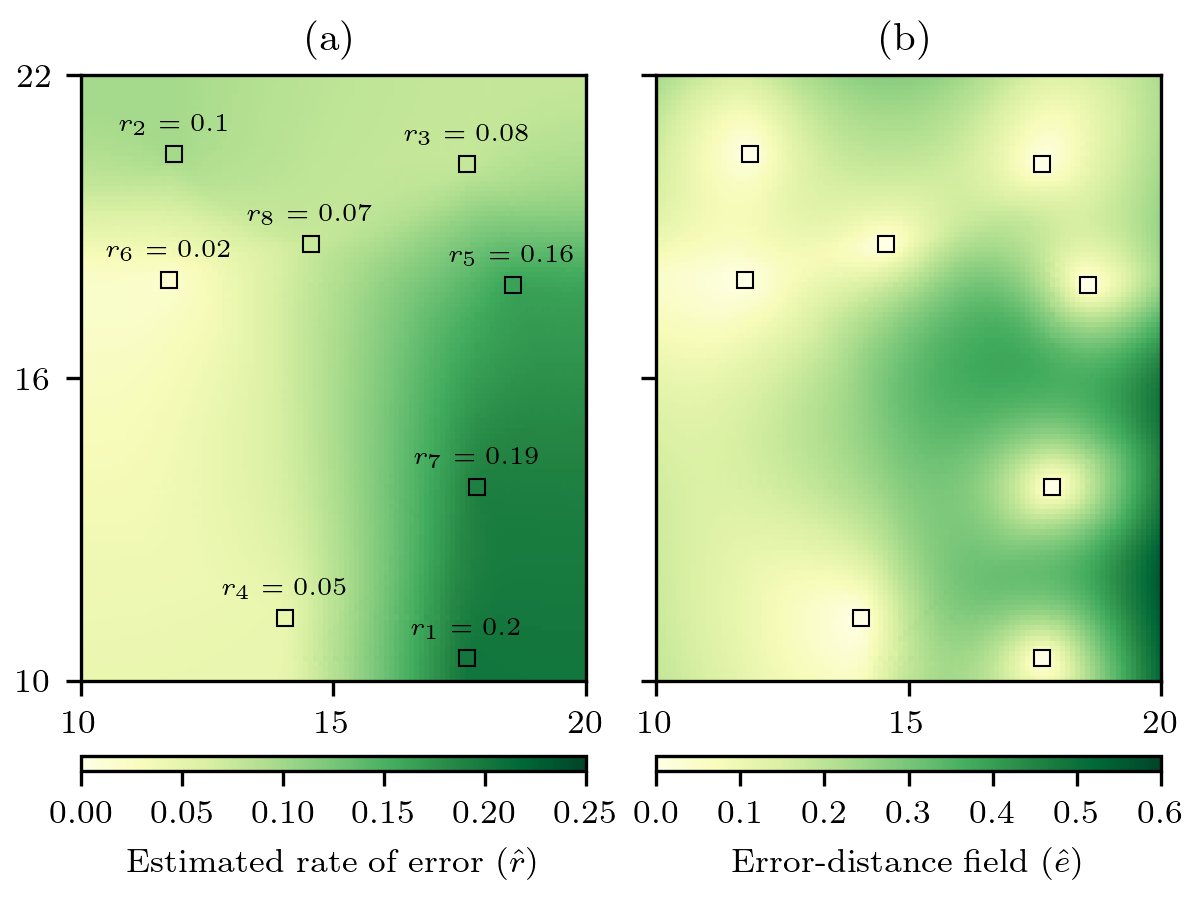

Supplement: Supplemental Information 1 [file peerj-cs-06-282-s001.zip › nni-discrete-method/figure-3.png]
